# Age-related association of aortic arch pulse wave velocity assessed by MRI with incident cardiovascular events: the multi-ethnic study of atherosclerosis (MESA)

**DOI:** 10.1186/1532-429X-18-S1-P132

**Published:** 2016-01-27

**Authors:** Yoshiaki Ohyama, Bharath Ambale Venkatesh, Chikara Noda, Jang-Young Kim, Yutaka Tanami, Gisela Teixido-Tura, Atul Chugh, Alban Redheuil, Chia-Ying Liu, Colin O Wu, Gregory Hundley, David A Bluemke, Joao A Lima

**Affiliations:** 1 grid.21107.350000000121719311Cardiology, Johns Hopkins University, Baltimore, MD USA; 2 grid.94365.3d0000000122975165National Institutes of Health, Bethesda, MD USA; 3 Wake Forest School of Medicine, Winston-Salem, MD USA; 4 Groupe Hospitalier La Pitié Salpêtrière Sorbonne Universités, Paris, France; 5 grid.411083.f0000000106758654Vall d'Hebron Hospital, Barcelona, Spain; 6 grid.15444.300000000404705454Yonsei University, Seoul, Korea (the Republic of; 7 Jewish Hospital, Luisville, KY USA; 8 National Heart, Lung and Blood Institute, Bethesda, MD USA

## Background

The carotid-femoral pulse wave velocity (PWV) assessed by tonometry is predictive of future cardiovascular disease (CVD) events; however, the predictive value of aortic arch PWV assessed by MRI for CVD events has not been established in the general population. The aim of the present study was to evaluate the association of arch PWV with incident CVD events over 10 years based on the Multi-Ethnic Study of Atherosclerosis (MESA).

## Methods

Aortic arch PWV was measured using through-plane aortic flow from phase contrast (PC) cine MRI at the level of the pulmonary artery bifurcation for transit time and black-blood sagittal images for transit length at baseline in 3,527 MESA participants free of overt CVD. Cox regression was used to evaluate the risk of incident CVD in relation to arch PWV adjusted for age, gender, race, and CV risk factors. Arch PWV were logarithmically transformed for COX regression models due to its right-skewed distribution (logPWV). There was significant interaction between arch PWV and age for outcomes, so analysis was repeated in each age decade (45-54, 55-64, 65-74, 75-84 years).

## Results

At baseline, participants were 62 ± 10 years of age; 53% women, 36% White, 15% Chinese, 29% African American, 20% Hispanic, and 45% had hypertension. The median value of arch PWV was 7.4 (IQR; 5.6 to 10.2) m/s. There were 427 CVD events over the 10-year follow-up. There was no significant association of PWV with incident CVD in all participants after adjustment for CVD risk factors. Stratifying by age groups, only 45-55-year-old participants had significant association of arch PWV with incident CVD in multivariable analysis (HR, 1.47; 95% confidence interval (CI), 1.10-1.97; p = 0.009), whereas other age groups did not (Table 1).

**Table 1:** Hazard Ratios of the logPWV for Cardiovascular Events Stratified by Age Groups no. of events

	no. of events	Unadjusted		Model 1		Model 2	
		HR (95% CI)	p	HR (95% CI)	p	HR (95% CI)	
All participants (n = 3,529)	427	1.26 (1.16-1.38)	<0.001	1.06 (0.97-1.17)	0.21	1.03 (0.94-1.14)	0.46
Age categories							
45-54 years old (n-1,027)	53	1.59 (1.23-2.06)	<0.001	1.48 (1.15-1.92)	0.002	1.47 (1.10-1.97)	0.009
55-64 years old (n-946)	96	1.10 (0.90-1.35)	0.35	1.08 (0.89-1.33)	0.43	0.99 (0.79-1.24)	0.94
65-75 years old (n-1,071)	169	1.05 (0.90-1.22)	0.51	1.03 (0.88-1.20)	0.75	1.01 (0.87-1.19)	0.87
75-84 years old (n = 485)	109	0.94 (0.78-1.14)	0.55	0.95 (0.78-1.15)	0.56	1.00 (0.83-1.22)	0.98

Hazard ratios are indicated per 1SD higher logPWV. Adjustment was performed for the following risk factors: model 1 = adjusted for age, gender, and race; model 2 = model 1 + mean blood pressure, antihypertensive medication use, diabetes, smoking, total cholesterol, HDL cholesterol, BMI.

HR indicates hazard ratio; BMI, body mass index; HDL, high density lipoprotein; PWV, pulse wave velocity; logPWV, log-transformed PWV

## Conclusions

Aortic arch PWV assessed by MRI is a significant predictor of CVD events among middle-age (45 to 54 years old) individuals, whereas arch PWV is not associated with CVD among elderly in a large multi-ethnic population.

